# Predicted vitamin D status and pancreatic cancer risk in two prospective cohort studies

**DOI:** 10.1038/sj.bjc.6605658

**Published:** 2010-04-13

**Authors:** Y Bao, K Ng, B M Wolpin, D S Michaud, E Giovannucci, C S Fuchs

**Affiliations:** 1 Channing Laboratory, Department of Medicine, Brigham and Women's Hospital, Harvard Medical School, 181 Longwood Avenue, Boston, MA 02115, USA; 2 Department of Adult Oncology, Dana-Farber Cancer Institute, Boston, MA, USA; 3 Department of Epidemiology, Harvard School of Public Health, Boston, MA, USA; 4 Department of Epidemiology and Public Health, Imperial College London,London, UK; 5 Department of Nutrition, Harvard School of Public Health, Boston, MA, USA

**Keywords:** vitamin D, pancreatic cancer, epidemiology

## Abstract

**Background:**

Studies evaluating vitamin D status in relation to pancreatic cancer risk have yielded inconsistent results.

**Methods:**

We prospectively followed 118 597 participants in the Nurses' Health Study and Health Professionals Follow-up Study from 1986 to 2006. We calculated a 25-hydroxyvitamin D (25(OH)D) score from known predictors of vitamin D status for each individual and then examined the predicted 25(OH)D levels in relation to pancreatic cancer risk. Relative risks (RRs) and 95% confidence intervals (95% CIs) were estimated using Cox proportional hazards models adjusted for age, sex, race, height, smoking, and diabetes. We then further adjusted for body mass index (BMI) and physical activity in a sensitivity analysis.

**Results:**

During 20 years of follow-up, we identified 575 incident pancreatic cancer cases. Higher 25(OH)D score was associated with a significant reduction in pancreatic cancer risk; compared with the lowest quintile, participants in the highest quintile of 25(OH)D score had an adjusted RR of 0.65 (95% CI=0.50–0.86; *P*_trend_=0.001). Results were similar when we further adjusted for BMI and physical activity.

**Conclusions:**

Higher 25(OH)D score was associated with a lower risk of pancreatic cancer in these two prospective cohort studies.

The role of vitamin D in carcinogenesis has attracted increasing attention in recent years. Certain observational studies have reported a protective association between sufficient vitamin D status and lower risk of colon, breast, prostate, and ovarian cancers (Garland *et al*, 2006). Experimental studies showed that vitamin D has anticancer properties, including inducing differentiation, initiating apoptosis, and inhibiting proliferation, angiogenesis, and metastasis (Giovannucci, 2005). Pancreatic cancer cells express vitamin D receptors (VDRs) (Colston *et al*, 1997) and high levels of 25(OH)D_3_-1*α*-hydroxylase, an enzyme that converts 25(OH)D to the active metabolite, 1,25-dihydroxyvitamin D (1,25(OH)_2_D) (Schwartz *et al*, 2004). In addition, 1,25 vitamin D analogs inhibit pancreatic cancer cell line growth *in vitro* (Colston *et al*, 1997; Schwartz *et al*, 2004) and pancreatic tumour growth *in vivo* (Colston *et al*, 1997). Therefore, vitamin D may have a role in the development of pancreatic cancer.

However, studies evaluating vitamin D status in relation to pancreatic cancer risk have yielded inconsistent results. To evaluate the impact of vitamin D status in a larger population, we developed a model to predict long-term plasma 25(OH)D levels. This 25(OH)D score takes into account the combined influence of many of the major determinants of vitamin D status. In previous analyses, the score was correlated with plasma 25(OH)D levels and was inversely associated with total cancer mortality and pancreatic cancer incidence in men (follow-up up to 2000; number of pancreatic cancer cases=170) (Giovannucci *et al*, 2006). In this study, we assessed the relation between predicted long-term vitamin D status and pancreatic cancer risk in both men and women and with additional follow-up (up to 2006), and further examined the potential effect modifications by other factors, using data collected from the Nurses' Health Study (NHS) and the Health Professionals Follow-up Study (HPFS).

## Materials and methods

In 1976, the NHS cohort was established when 121 700 US female registered nurses aged 30–55 years answered a baseline questionnaire on risk factors for cancer and cardiovascular disease. Every 2 years, participants receive follow-up questionnaires to update information on potential risk factors and new cancer and disease diagnoses. Dietary information was first collected in 1980 through a food frequency questionnaire, and is updated in alternate follow-up cycles; leisure-time physical activity was assessed in the 1986 questionnaire. Blood samples were provided by 32 826 participants aged 43–70 years from 1989 to 1990.

In 1986, the HPFS cohort was established when 51 529 male dentists, optometrists, osteopaths, podiatrists, pharmacists, and veterinarians aged 40–75 years responded to a baseline questionnaire on risk factors for cancer, cardiovascular disease, and diabetes. A follow-up questionnaire is sent to participants every 2 years requesting an update on nondietary exposures and medical history, with dietary history updated every 4 years. Blood samples were provided by 18 018 participants from 1993 to 1995.

The overall follow-up rate was over 90% in both cohorts. For the present analysis, we excluded individuals with a history of cancer up to 1986, leaving a cohort of 118 597 participants eligible (45 226 men and 73 371 women). This study was approved by the human subjects committee at Brigham and Women's Hospital and the Harvard School of Public Health. All participants provided informed consent for questionnaire and blood data to be used in research studies.

In both cohorts, when a participant (or next of kin for decedents) reported a diagnosis of pancreatic cancer on a follow-up questionnaire, we asked permission to obtain the participant's medical records. We also searched the National Death Index to identify deaths among non-respondents. If the primary cause of death on the death certificate was a previously unreported pancreatic cancer case, we contacted a family member to obtain permission to retrieve medical records. Study physicians at the NHS and HPFS who were masked to the exposure data reviewed medical records and assigned cancer diagnoses and causes of death. In this study, 90% of the pancreatic cancer cases were confirmed by medical record review.

Derivation of the 25(OH)D prediction score has been described previously (Giovannucci *et al*, 2006). Briefly, we used a sample of 1095 men in the HPFS who had available plasma 25(OH)D measurements and who were free of diagnosed cancer at the time of blood draw. With the measured plasma 25(OH)D concentration as the dependent variable, linear regression was performed using race, geographic region, vitamin D intake, body mass index (BMI), and leisure-time physical activity as independent predictors of plasma 25(OH)D. Then, on the basis of the predictors' regression coefficients from the sample, a 25(OH)D score was calculated for each cohort member. To validate this model, we calculated the 25(OH)D score for an independent sample of 542 men in the HPFS who also had available measurements of circulating 25(OH)D (Giovannucci *et al*, 2006). The actual plasma concentration rose across increasing deciles of 25(OH)D score (*P*_trend_ <0.001), and the difference in the mean actual 25(OH)D concentration between extreme deciles was 10 ng ml^−1^, similar to the difference of 11 ng ml^−1^, which we calculated from the initial dataset (1 ng ml^−1^=2.496 nmol l^−1^). In addition, in a separate analysis of 47 800 men, 25(OH)D score had a significantly inverse association with colorectal cancer, which is compatible with plasma-based studies (Giovannucci *et al*, 2006). We then applied this method to women and calculated 25(OH)D score for all the participants in our study cohort using baseline race and geographic region, and values of leisure-time physical activity, BMI, and vitamin D intake reported in the 1986 questionnaire (when leisure-time physical activity was first assessed).

We collected information on height, race, and geographic regions from the 1986 HPFS questionnaire and the 1976 NHS questionnaire, the only questionnaires in which these variables were assessed. BMI (kg m^−2^) was calculated using height (reported in 1986 for HPFS and in 1976 for NHS) and weight was reported in the 1986 questionnaires for both cohorts. Information on cigarette smoking, physical activity, history of diabetes, multivitamin use, and dietary factors was also obtained from the 1986 questionnaires for both cohorts.

### Statistical analyses

We computed person-time from the return date of the 1986 questionnaire to the date of pancreatic cancer diagnosis, death from any cause, or the end of follow-up (30 June 2006 in the NHS, and 31 January 2006 in the HPFS), whichever came first. Relative risks (RRs) and 95% confidence intervals (CIs) were estimated by Cox proportional hazards models with age (in years) as the primary time scale. The proportional hazards assumption was verified by modelling interaction terms of age and our main exposures as well as other fixed covariates. We first analysed the HPFS and the NHS separately; and if similar RRs were obtained, we would then perform a pooled analysis with adjustment for cohort. 25-Hydroxyvitamin D score was analysed in quintiles based on predicted levels, with the lowest quintile as the reference group. Quintiles were based on the distribution observed in the entire study population. In multivariate models, we adjusted for sex, race (Caucasian, African American, other), height (inches in quintiles), cigarette smoking (never, past, current <15 cigarettes per day, current 15–35 cigarettes per day, current >35 cigarettes per day), and diabetes (yes/no). We considered the possibility that the 25(OH)D score might be acting as a surrogate for other risk factors of pancreatic cancer, such as BMI or physical activity, which were both in the prediction equation. We therefore further adjusted for BMI (kg m^−2^ in quintiles) and physical activity (MET-h per week in quintiles) in the multivariate model. An indicator variable for missing values of each covariate was created. Linear trends were tested by the Wald test of a score variable that contained median values of 25(OH)D quintiles.

We performed subgroup analyses to examine whether the association of 25(OH)D score with pancreatic cancer varies across strata of sex (also across strata of study, as HPFS consisted of only men and NHS of only women), age at baseline (<60 or ⩾60 years), follow-up duration (<10 or ⩾10 years), geographic region (northern or southern), smoking (never or ever), BMI (<25 or ⩾25 kg m^−2^), physical activity (below or above median, 8.7 MET-h per week), dietary vitamin D intake (below or above median, 288 IU per day), calcium intake (below or above median, 888 mg per day), multivitamin use (no or yes), and use of supplemental vitamin D (no or yes). We also examined whether retinol intake (below or above median, 2669 IU per day) modified the association between 25(OH)D score and pancreatic cancer because retinol can compete with vitamin D for binding to the retinoid X receptor (Giovannucci, 2005). Tests for interaction were performed by the Wald test using cross-product term of predicted 25(OH)D quintile trend with the stratification variables.

In sensitivity analyses, we excluded the first 2 years of follow-up for all participants to rule out an effect of subclinical pancreatic cancer on 25(OH)D levels. We also did a subgroup analysis including only Caucasians. For analyses among men or women, we repeated our analyses using sex-specific quintiles, which yielded consistent results with those based on quintiles generated from the entire population. Excluding cases with missing medical records (10% of all cases) had no impact on the estimates. All analyses used SAS software, version 9.1 (SAS Institute, Cary, NC, USA).

## Results

With 2 226 169 person-years accrued over 20 years of follow-up, a total of 575 pancreatic cancer cases were identified (273 men and 302 women). The median predicted 25(OH)D level was 29.2 ng ml^−1^ in men and 27.6 ng ml^−1^ in women. Participants with higher 25(OH)D score were more likely to be Caucasian, live in southern states, have a lower BMI, report higher physical activity, have higher vitamin D intake, and were less likely to be current smokers or have a history of diabetes (Table 1).

We pooled the HPFS and the NHS together because their designs were similar and no significant differences in the risk estimates were found when the two studies were analysed separately (Table 3, the stratified analysis by sex). Higher 25(OH)D score was associated with a significant reduction in risk (Table 2). Compared with individuals with 25(OH)D scores in the lowest quintile, those in the highest quintile had an adjusted RR of 0.65 (95% CI=0.50–0.86; *P*_trend_=0.001) for pancreatic cancer. This result remained largely unchanged after further adjusting for BMI and physical activity or after excluding the first 2 years of follow-up (Table 2). Restricting to Caucasians had little impact on the estimates (data not shown).

Associations with 25(OH)D score were examined across strata of other factors (Table 3). No statistically significant interactions were found; higher predicted 25(OH)D remained inversely related to risk across most subgroups. We noted that there was a trend towards a greater impact of higher 25(OH)D scores on risk among participants of older age, southern region, nonsmokers, low physical activity, low dietary vitamin D intake, low retinol intake, no multivitamin use, and no supplemental vitamin D use (Table 3).

## Discussion

In our study, higher 25(OH)D score was associated with a significantly lower risk of pancreatic cancer. This inverse association was consistent across gender and strata of other covariates. There are several mechanisms through which vitamin D may affect pancreatic cancer risk. Pancreatic cancer cells express VDRs (Colston *et al*, 1997) and 25(OH)D_3_-1a-hydroxylase (Schwartz *et al*, 2004), which metabolises 25(OH)D to the active 1,25(OH)_2_D vitamin D form. Binding of VDRs by 1,25(OH)_2_D leads to increased differentiation and apoptosis as well as reduced proliferation, invasiveness, angiogenesis, and metastasis (Giovannucci, 2005); and experimental studies have shown that 1,25(OH)_2_D analogs inhibit growth of pancreatic cancer cells *in vitro* and *in vivo* (Colston *et al*, 1997; Schwartz *et al*, 2004). In addition, pancreatic islet cells express VDRs and 25(OH)D_3_-1a-hydroxylase, and *in vitro* and *in vivo* evidence supports that vitamin D deficiency impairs endocrine pancreatic function (Pittas *et al*, 2007). Observational studies have shown that vitamin D status is inversely associated with development of type 2 diabetes or metabolic syndrome (Mattila *et al*, 2007; Pittas *et al*, 2007; Forouhi *et al*, 2008). As diabetes, hyperglycaemia, and insulin resistance have been linked to pancreatic cancer development, vitamin D may act to decrease pancreatic cancer risk by improving glucose metabolism and reducing insulin resistance.

Epidemiological studies have used four approaches to examine the association of pancreatic cancer with vitamin D status, and their results have been inconsistent. Sunlight exposure, a major source of vitamin D in humans, was inversely correlated with pancreatic cancer in ecological studies conducted in North America, Europe, and Japan (Mizoue, 2004; Boscoe and Schymura, 2006; Grant, 2007). Higher dietary vitamin D intake as well as higher total vitamin D intake (from foods and supplements) was related to lower risk in the NHS and the HPFS (Skinner *et al*, 2006) but not in a cohort of male Finnish smokers enrolled in the Alpha-Tocopherol, Beta-Carotene (ATBC) Cancer Prevention Study (Stolzenberg-Solomon *et al*, 2002). Directly measured circulating 25(OH)D, which reflects vitamin D status from both sun and dietary sources, was positively associated with pancreatic cancer risk in male Finnish smokers (fifth *vs* first quintile, RR=2.92, 95% CI=1.56–5.48) (Stolzenberg-Solomon *et al*, 2006) but not in a cohort of the Prostate, Lung, Colorectal, and Ovarian (PLCO) Screening Trial (fifth *vs* first quintile, RR=1.45, 95% CI=0.66–3.15) (Stolzenberg-Solomon *et al*, 2009). In contrast, the vitamin D prediction score, which also accounts for sun exposure (by using residential state and physical activity as surrogates) and vitamin D intake, was inversely associated with pancreatic cancer risk in the present two US prospective cohort studies (NHS and HPFS) (Giovannucci *et al* (2006) and this study).

However, participants in the ATBC study might not be comparable with those in the American studies, as they were all current smokers and lived at higher latitude. In this study, we observed a stronger inverse association with vitamin D among nonsmokers and among those living in southern states. The PLCO study also found a significant interaction by geographic region (*P*_interaction_=0.015): they reported a positive association of risk with plasma vitamin D concentrations among those living in northern latitudes, but no association was observed among those living in southern latitudes (Stolzenberg-Solomon *et al*, 2009). Another difference in the ATBC study population concerns dietary pattern: a major dietary source of vitamin D in the Finns does not tend to be from fortified dairy products or breakfast cereal as American populations but from vitamin D-rich fish that may contain some pancreatic carcinogen such as organochlorine compounds (Stolzenberg-Solomon *et al*, 2006). In addition, both the ATBC study and the PLCO study were based on one measurement of 25(OH)D in blood, which most likely reflects recent exposure to sources of vitamin D rather than long-term average vitamin D level; whereas the 25(OH)D prediction score would track well over time because factors that influence the score, such as race and residential region, are immutable or relatively stable. As evidence of this supposition, in a previous study, the correlation between two direct plasma measurements 4 years apart was 0.70, whereas the correlation between the two 25(OH)D scores was 0.83 (Giovannucci *et al*, 2006).

Retinol has been hypothesised to counteract the cancer prevention effects of vitamin D, possibly acting through competition with vitamin D for the retinoid X receptor (Giovannucci, 2005). In this study, we observed that the inverse association between vitamin D status and pancreatic cancer was more pronounced among those with lower intake of retinol; we also observed a stronger inverse association among those who did not use multivitamins or supplemental vitamin D. We have previously reported an increased pancreatic cancer risk associated with multivitamin use (Skinner *et al*, 2004), so it is possible that some factor in multivitamin supplements other than vitamin D, potentially retinol, antagonises the protective effect of vitamin D or increases risk independently.

One concern of our approach is that the 25(OH)D score may act as a surrogate for other potential risk factors, such as BMI or physical activity. A higher BMI and less physical activity have previously been associated with increased pancreatic cancer risk in our cohorts (Michaud *et al*, 2001). However, our results for predicted 25(OH)D did not change when adjusted for BMI or physical activity, which suggests that total vitamin status, rather than simply low BMI or physical activity, is driving the significant inverse association. We are also aware that the prediction score has been developed in men; however, given the prospective design, any misclassification of the 25(OH)D score among women tended to be non-differential, and therefore would only bias the results towards the null. In addition, when we stratified the analyses by gender, similar RRs were obtained for men and women, with no significant interactions. To rule out the possibility that vitamin D status might be changed by preclinical pancreatic cancer at baseline, we excluded the first 2 years of follow-up for all participants in sensitivity analyses. The results were unchanged. Residual confounding by smoking was not likely because a stronger association was observed among never smokers. Residual confounding by other measured factors might be of minor importance in this study, as our age/sex-adjusted models and multivariate models yielded very similar results.

The strengths of our study include its prospective design, use of a validated prediction score taking into account both diet and sun exposure, comprehensive information on many potential confounders, a large sample size that allowed us to stratify the data by potential effect modifiers, and 20 years of follow-up with a high follow-up rate. Because the participants were health professionals, the accuracy of self-reported data is likely to be high; moreover, any misclassification of vitamin D status is likely to be random and would therefore have attenuated rather than exaggerated a true association.

In conclusion, our data suggest that higher 25(OH)D levels may significantly decrease the risk of pancreatic cancer. Given the growing epidemic of vitamin D insufficiency in the US population (Ginde *et al*, 2009), more epidemiological research is needed, particularly prospective studies with repeated measures of plasma 25(OH)D. If the association between vitamin D and pancreatic cancer is causal, many with low vitamin D levels might benefit from increased vitamin D status for pancreatic cancer risk reduction.

## Figures and Tables

**Table 1:** Baseline characteristics by predicted plasma 25(OH)D, 1986

	**Predicted plasma 25(OH)D^a^**				
**Characteristic**	**Quintile 1**	**Quintile 2**	**Quintile 3**	**Quintile 4**	**Quintile 5**
*N*	23 670	23 840	23 643	23 673	23 771
Range 25(OH)D (ng ml^−1^)	7.3–25.2	25.2–27.4	27.4–29.1	29.1–31.1	31.2–38.6
Median 25(OH)D (ng ml^−1^)	23.4	26.4	28.3	30.0	32.6
Mean age (years)	52.7	53.1	53.3	53.5	53.6
					
*Race* (%)					
White	88.70	94.75	95.32	96.07	96.36
Black	4.87	0.42	0.14	0.02	0
Other	6.43	4.83	4.54	3.91	3.64
					
*Residence* (%)					
North-east	61.5	51.5	49.4	41.0	37.4
Mid-west/west	28.3	27.8	26.6	26.9	25.3
South	10.2	20.7	24.0	32.1	37.3
Mean height (inches)	65.2	66.5	66.8	67.4	67.3
Mean body mass index (kg m^−2^)	29.1	26.2	24.6	24.1	23.1
Median physical activity (MET-h per week)	5.8	9.0	13.6	20.1	34.5
					
*Smoking* (%)					
Current	20.4	18.3	17.5	14.2	12.3
Past	35.0	37.9	38.3	40.0	39.8
Never	44.6	43.8	44.2	45.8	47.9
Diabetes (%)	5.8	3.9	2.9	2.9	2.3
Mean vitamin D intake (IU per day)	200.8	288.8	343.2	431.0	570.2

Abbreviations: 25(OH)D=25-hydroxyvitamin D; MET=metabolic equivalents; h=hour; IU=international units.

a Quintiles were generated from the Nurses' Health Study and the Health Professionals Follow-Up Study combined.

**Table 2:** RRs and 95% CIs of pancreatic cancer by predicted plasma 25(OH)D, 1986–2006

	**Predicted plasma 25(OH)D^a^**					
	**Quintile 1**	**Quintile 2**	**Quintile 3**	**Quintile 4**	**Quintile 5**	***P*_trend_ b**
Range 25(OH)D (ng ml^−1^)	7.3–25.2	25.2–27.4	27.4–29.1	29.1–31.1	31.2–38.6	
Median 25(OH)D (ng ml^−1^)	23.4	26.4	28.3	30.0	32.6	
Cases (*n*=575)	125	121	115	112	102	
Person-years	444 248	445 340	443 622	443 944	449 015	
Age/sex-adjusted	1.00	0.84 (0.66–1.09)	0.77 (0.59–1.00)	0.71 (0.54–0.92)	0.63 (0.48–0.83)	<0.001
Multivariate^c^	1.00	0.85 (0.65–1.09)	0.77 (0.59–1.00)	0.72 (0.55–0.94)	0.65 (0.50–0.86)	0.001
Multivariate+BMI+activity^d^	1.00	0.85 (0.65–1.11)	0.77 (0.57–1.04)	0.72 (0.52–1.00)	0.67 (0.46–0.96)	0.03
Multivariate+BMI+activity, excluding the first 2 years of follow-up^e^	1.00	0.85 (0.65–1.12)	0.76 (0.56–1.04)	0.69 (0.50–0.97)	0.66 (0.46–0.96)	0.02

Abbreviations: 25(OH)D=25-hydroxyvitamin D; BMI=body mass index; CI=confidence interval; RR=relative risk.

a Quintiles were generated from the Nurses' Health Study and the Health Professionals Follow-Up Study combined.

b Calculated by using median predicted plasma 25(OH)D as a continuous variable.

c Adjusted for age, sex (cohort), race (Caucasian, African American, other), height (inches in quintiles), cigarette smoking (never, past, current <15 cigarettes per day, current 15–35 cigarettes per day, current >35 cigarettes per day), and diabetes (yes or no).

d In addition, adjusted for body mass index (kg m^−2^ in quintiles) and physical activity (MET-h per week in quintiles).

e Cases/person-years: 556/1 989 401.

**Table 3:** RRs and 95% CIs of pancreatic cancer by predicted plasma 25(OH)D according to other factors, 1986–2006

		**Predicted plasma 25(OH)D^a^**						
	**Cases**	**Quintile 1**	**Quintile 2**	**Quintile 3**	**Quintile 4**	**Quintile 5**	***P*_trend_ b**	***P*_interaction_ c**
*Sex*								0.75
Male (HPFS)	273	1.00	0.88 (0.55–1.42)	0.71 (0.43–1.19)	0.64 (0.37–1.11)	0.66 (0.35–1.21)	0.12	
Female (NHS)	302	1.00	0.79 (0.56–1.13)	0.81 (0.55–1.21)	0.80 (0.52–1.24)	0.64 (0.39–1.04)	0.10	
								
*Age at baseline (years)*								0.41
<60	298	1.00	0.76 (0.52–1.10)	0.83 (0.55–1.24)	0.78 (0.50–1.22)	0.76 (0.46–1.26)	0.34	
⩾60	277	1.00	0.93 (0.63–1.37)	0.70 (0.45–1.08)	0.65 (0.41–1.05)	0.57 (0.33–0.97)	0.02	
								
*Follow-up (years)*								0.60
<10	225	1.00	1.01 (0.66–1.55)	0.70 (0.43–1.16)	0.85 (0.50–1.43)	0.68 (0.37–1.25)	0.19	
⩾10	350	1.00	0.75 (0.53–1.06)	0.81 (0.56–1.17)	0.65 (0.43–0.99)	0.66 (0.41–1.05)	0.07	
								
*Region*								0.62
Northern	289	1.00	0.82 (0.57–1.18)	0.97 (0.65–1.44)	0.94 (0.60–1.47)	0.83 (0.49–1.41)	0.65	
Southern	286	1.00	0.90 (0.59–1.37)	0.63 (0.39–1.01)	0.61 (0.37–1.01)	0.58 (0.33–1.01)	0.03	
								
*Smoking*								0.16
Never	222	1.00	0.81 (0.52–1.24)	0.73 (0.45–1.17)	0.65 (0.39–1.10)	0.40 (0.22–0.74)	0.01	
Ever	353	1.00	0.86 (0.61–1.22)	0.79 (0.54–1.16)	0.75 (0.50–1.15)	0.88 (0.56–1.40)	0.50	
								
*Body mass index (kg m*^−*2*^)								0.77
<25	271	1.00	0.76 (0.42–1.38)	0.82 (0.46–1.47)	0.70 (0.38–1.28)	0.57 (0.31–1.07)	0.05	
⩾25	304	1.00	0.87 (0.64–1.20)	0.69 (0.47–1.03)	0.69 (0.44–1.09)	0.83 (0.47–1.49)	0.14	
								
*Physical activity (MET-h per week)*								0.58
<8.7 (median)	305	1.00	0.82 (0.60–1.12)	0.73 (0.51–1.06)	0.73 (0.48–1.13)	0.40 (0.20–0.80)	0.01	
⩾8.7	270	1.00	0.95 (0.54–1.66)	0.89 (0.51–1.56)	0.81 (0.46–1.43)	0.86 (0.47–1.55)	0.58	
								
*Dietary vitamin D intake (IU per day)*								0.67
<288 (median)	277	1.00	0.64 (0.45–0.91)	0.58 (0.38–0.89)	0.38 (0.22–0.65)	0.34 (0.17–0.67)	<0.01	
⩾288	298	1.00	0.87 (0.53–1.42)	0.69 (0.41–1.17)	0.78 (0.45–1.35)	0.71 (0.38–1.30)	0.30	
								
*Retinol (IU per day)*								0.23
<2669 (median)	280	1.00	0.70 (0.48–1.00)	0.65 (0.43–1.00)	0.55 (0.34–0.91)	0.42 (0.23–0.78)	0.01	
⩾2669	295	1.00	0.86 (0.56–1.33)	0.71 (0.44–1.13)	0.71 (0.43–1.16)	0.75 (0.43–1.29)	0.29	
								
*Calcium (mg per day)*								0.29
<888 (median)	307	1.00	0.85 (0.59–1.21)	0.86 (0.57–1.28)	0.65 (0.41–1.04)	0.76 (0.44–1.30)	0.19	
⩾888	268	1.00	0.81 (0.53–1.24)	0.65 (0.41–1.04)	0.75 (0.47–1.22)	0.59 (0.34–1.02)	0.08	
								
*Multivitamin use*								0.77
No	335	1.00	0.75 (0.54–1.05)	0.61 (0.42–0.91)	0.56 (0.36–0.88)	0.42 (0.23–0.74)	<0.01	
Yes	240	1.00	0.87 (0.52–1.46)	0.89 (0.52–1.52)	0.82 (0.47–1.45)	0.89 (0.48–1.64)	0.78	
								
*Supplemental vitamin D use*								0.95
No	346	1.00	0.72 (0.52–1.00)	0.59 (0.40–0.87)	0.59 (0.38–0.92)	0.47 (0.26–0.85)	<0.01	
Yes	229	1.00	1.02 (0.57–1.83)	1.03 (0.56–1.88)	0.85 (0.45–1.61)	0.86 (0.43–1.72)	0.50	

Abbreviations: 25(OH)D=25-hydroxyvitamin D; MET=metabolic equivalents; h=hour; IU=international units; CI=confidence interval; RR=relative risk.

When applicable, adjusted for age, sex (cohort), race (Caucasian, African American, other), height (inches in quintiles), body mass index (kg m^−2^ in quintiles), physical activity (MET-h per week in quintiles), cigarette smoking (never, past, current <15 cigarettes per day, current 15–35 cigarettes per day, current >35 cigarettes per day), and diabetes (yes or no).

a Quintiles were generated from the Nurses' Health Study and the Health Professionals Follow-Up Study combined.

b Calculated by using median predicted plasma 25(OH)D as a continuous variable.

c Calculated by Wald test using cross-product term of predicted plasma 25(OH)D quintile trend with the stratification variables.
